# Global vulnerability of soil ecosystems to erosion

**DOI:** 10.1007/s10980-020-00984-z

**Published:** 2020-03-10

**Authors:** Carlos A. Guerra, Isabel M. D. Rosa, Emiliana Valentini, Florian Wolf, Federico Filipponi, Dirk N. Karger, Alessandra Nguyen Xuan, Jerome Mathieu, Patrick Lavelle, Nico Eisenhauer

**Affiliations:** Institute of Biology, Martin Luther University Halle-Wittenberg, Am Kirchtor 1, 06108 Halle (Saale), Germany; German Centre for Integrative Biodiversity Research (iDiv), Halle-Jena-Leipzig, Deutscher Platz 5E, 04103 Leipzig, Germany; School of Natural Sciences, Bangor University, Gwyned, Wales, UK; Institute for Environmental Protection and Research (ISPRA), Via Vitaliano Brancati 48, 00144 Roma, Italy; Institute of Biology, Martin Luther University Halle-Wittenberg, Am Kirchtor 1, 06108 Halle (Saale), Germany; German Centre for Integrative Biodiversity Research (iDiv), Halle-Jena-Leipzig, Deutscher Platz 5E, 04103 Leipzig, Germany; Institute for Environmental Protection and Research (ISPRA), Via Vitaliano Brancati 48, 00144 Roma, Italy; Institute of Systematic Botany, University of Zurich, Zollikerstrasse 107, 8008 Zurich, Switzerland; Swiss Federal Research Institute WSL, Zürcherstrasse 111, 8903 Birmensdorf, Switzerland; Institute for Environmental Protection and Research (ISPRA), Via Vitaliano Brancati 48, 00144 Roma, Italy; Sorbonne Université, CNRS, UPEC, Paris 7, INRA, IRD, Institut d’Ecologie et des Sciences de l’Environnement de Paris, 75005 Paris, France; German Centre for Integrative Biodiversity Research (iDiv), Halle-Jena-Leipzig, Deutscher Platz 5E, 04103 Leipzig, Germany; Institute of Biology, Leipzig University, Deutscher Platz 5e, 04103 Leipzig, Germany

**Keywords:** Soil erosion, Soil protection, Temporally explicit, Belowground biodiversity, Ecosystem service supply, Mapping

## Abstract

**Context:**

Soil erosion is one of the main threats driving soil degradation across the globe with important impacts on crop yields, soil biota, biogeochemical cycles, and ultimately human nutrition.

**Objectives:**

Here, using an empirical model, we present a global and temporally explicit assessment of soil erosion risk according to recent (2001–2013) dynamics of rainfall and vegetation cover change to identify vulnerable areas for soils and soil biodiversity.

**Methods:**

We used an adaptation of the Universal Soil Loss Equation together with state of the art remote sensing models to create a spatially and temporally explicit global model of soil erosion and soil protection. Finally, we overlaid global maps of soil biodiversity to assess the potential vulnerability of these soil communities to soil erosion.

**Results:**

We show a consistent decline in soil erosion protection over time across terrestrial biomes, which resulted in a global increase of 11.7% in soil erosion rates. Notably, soil erosion risk systematically increased between 2006 and 2013 in relation to the baseline year (2001). Although vegetation cover is central to soil protection, this increase was mostly driven by changes in rainfall erosivity. Globally, soil erosion is expected not only to have an impact on the vulnerability of soil conditions but also on soil biodiversity with 6.4% (for soil macrofauna) and 7.6% (for soil fungi) of these vulnerable areas coinciding with regions with high soil biodiversity.

**Conclusions:**

Our results indicate that an increasing proportion of soils are degraded globally, affecting not only livelihoods but also potentially degrading local and regional landscapes. Similarly, many degraded regions coincide with and may have impacted high levels of soil biodiversity.

## Introduction

The role of soils in the supply of key ecosystem services is widely recognised (Wall et al. 2012; Gardi et al. 2013; Adhikari and Hartemink 2016; Baveye et al. 2016). Yet spatially-explicit assessments that globally depict the different processes contributing to soil-driven ecosystem services are still missing (Costanza et al. 2017). This lack of globally available information is even more pronounced when addressing soil biodiversity interactions. In this context, the Intergovernmental science-policy Platform on Biodiversity and Ecosystem Services (IPBES), while developing its regional and global assessments, is calling for researchers to actively contribute to assess the state and trends of biodiversity and ecosystem services supply (Perrings et al. 2010, 2011; Díaz et al. 2018). It also identifies soil biodiversity and soil ecosystem services as one of the major gaps in the current assessments. Additionally, following major global assessments in land degradation [e.g., the recent IPBES Assessment Report on Land Degradation and Restoration (IPBES 2018)], there is an important focus on halting land degradation in order to fulfil the Sustainable Development Goal 15 (https://sustainabledevelopment.un.org/sdg15), among others.

Soil erosion is one of the main threats driving soil degradation across the globe (Lal 2001; Zhang et al. 2010; Panagos et al. 2015a, b; Montanarella et al. 2016). Specifically, soil erosion has been shown to accentuate and be driven by the impacts of land-use and climate change (Lal 2003; Chappell et al. 2015; Paustian et al. 2016), to degrade soil conditions for biodiversity (Veresoglou et al. 2015; Wall et al. 2015), and to negatively influence biogeochemical cycles (Quinton et al. 2010). According to several climate and land use change studies (IPCC 2007; Hurtt et al. 2011; Guiot and Cramer 2016), soil erosion is reported to be increasing, resulting in a major threat to soil conditions and soil ecological processes (e.g., litter decomposition, nutrient cycling; FAO and ITPS 2015). While soil erosion can include several different processes, e.g., water erosion, wind erosion, freeze–thaw erosion, gravity erosion; here we focus on the effects of water erosion. Globally, soil erosion by water accounts for the greatest loss of soil directly associated with other global change drivers, like land use (e.g., clear-cutting, intensification of farming practices) and climate change (Yang et al. 2003; Borrelli et al. 2017), and significantly contributes to the reduction of several soil-related societal benefits (Wall and Six 2015; Adhikari and Hartemink 2016). In face of these anthropogenic landscape alterations, it is crucial to understand how to design, conserve, and manage our landscapes to sustainably provide ecosystem services that are essential for supporting human well-being now and into the future (Qiu et al. 2018).

Combined, these drivers of ecosystem change (i.e., climate, land use, land degradation) contribute to the degradation of soil conditions for many human livelihoods (Jónsson and Davídsdóttir 2016) and soil biodiversity (Gardi et al. 2013; Bardgett and van der Putten 2014). In view of this, recent assessments and meta-analyses (IPBES 2018) have established a relevant positive link between soil degradation and soil biodiversity declines. Nevertheless, the global vulnerability of soil biodiversity to soil degradation processes (i.e., the potential susceptibility of soil communities to erosion) is understudied, with current belowground conservation strategies focussing mainly on ecosystem processes (e.g., carbon sequestration) without a representation of how belowground diversity links to them (Nielsen et al. 2015).

Supported by a growing scientific literature (Seppelt et al. 2011; Costanza and Kubiszewski 2012; Costanza et al. 2014; Bennett et al. 2015; Orgiazzi and Panagos 2018), several initiatives underline the need for more consistent methodological approaches to globally quantify and map indicators of ecosystem service supply (Müller and Burkhard 2012; Guerra et al. 2016a) that are sensitive to policy and management impacts (Maes et al. 2012; Dunbar et al. 2013; Guerra et al. 2016b). Understanding and quantifying these ecosystem services (Díaz et al. 2018) relies on the availability of spatially and temporally explicit datasets of ecosystem service supply (Maes et al. 2013, 2015). From national to global scales, several policy initiatives (e.g., the Convention of Biological Diversity, the Sustainable Development Goals) depend on these datasets to evaluate the fulfilment of multiple nature conservation and sustainable development goals (Geijzendorffer et al. 2017). Nevertheless, many previous global or regional soil erosion risk assessments (here characterized as the ratio of change between the erosion rate in moment one and moment two) omit the quantification of the direct contribution of natural systems to the prevention of soil erosion or treat this process as static, overlooking long-term or inter-annual variations. In addition to overlooking the multiple spatial and temporal dimensions of soil erosion risk, these assessments also neglect the potential spatial matches (vulnerability areas) between erosion risk and soil biodiversity, particularly at the global scale.

Under the same environmental and climatic conditions, an increase in the amount of vegetation cover leads to a decrease in the risk of water driven soil erosion and, therefore, to a higher ecosystem service supply (Guerra et al. 2016a). In the current context where process-based physical models and the availability of input data are not yet mature enough for global scale applications (Yang et al. 2003; García-Ruiz et al. 2015), the use of physical empirical methods for predicting soil erosion risk can provide reasonably accurate estimates (Borrelli et al. 2017). These empirical models allow users to dynamically account for the effects of climate and land cover change by continuously modelling changes in rainfall erosivity and vegetation cover, respectively.

In contrast to previous applications, here we modelled the effects of rainfall erosivity and vegetation cover on global soil erosion rates, providing a global and temporally-explicit assessment of soil erosion protection for the period between 2001 and 2013. The temporal range was limited to this time period to minimize uncertainty errors coming from the different temporal scopes and modelling approaches of the underlying datasets used in the model. This resulted in a monthly evaluation of soil erosion protection, that allowed the identification and description of global patterns of soil erosion risk and soil erosion protection as well as vulnerable areas [here described as the degree to which a system is susceptible to soil erosion (De Lange et al. 2010)] where conservation strategies could have the most impact in halting soil degradation. Insight into the potential impacts on soil biodiversity was gained by comparing the changes in soil erosion risk with the global distribution of soil fungi and soil macrofauna obtained from previous global assessments (Tedersoo et al. 2014; Orgiazzi et al. 2016). These two soil biodiversity groups were selected in order to represent (i) soil organisms with substantially different size (Decaëns 2010), and (ii) organisms that drive crucial ecosystem processes like litter decomposition or soil respiration (Bardgett and van der Putten 2014), thus having significant feedback effects on soil erosion control (Lehmann et al. 2017).

## Methods

### General approach

The study area covers 91 Mkm^2^ including all major biogeographic regions of the world and most of the global land masses except the Arctic, the Antarctic, the Sahara Desert, Greenland, and urban surface areas. These exceptions mainly relate to limitations of the datasets used for the estimation of risk prevention (Table 1) and to the exclusion of urban areas, water surfaces and areas with permanent ice that fall outside the scope of this work.

Soil erosion protection is here defined as the amount of soil that is prevented from being eroded by water through the influence and erosion mitigation capacity of available vegetation (Guerra et al. 2014). Soil erosion protection comprises several processes, including natural protection and land use mitigation measures that happen at different scales and moments in time (Podmanicky et al. 2011; Baveye et al. 2016). Here we focus on two different aspects of soil erosion protection: (i) on vegetation cover dynamics and patterns, assuming that these encompass the amplitude of land use interactions that influence natural soil protection, and (ii) on the global ratio between the modelled soil erosion risk and the potential soil erosion risk. While the latter is equal to the fraction of vegetation cover for any given pixel [as all other factors are already accounted for in the different equation parameters (Eqs. 1 and 3)], when calculated globally it produces a weighted average that gives more relevance to places with higher soil erosion risk.

We acknowledge that other factors play important roles in the process of soil erosion prevention (e.g., terrain situation, soil flora and fauna, functional traits like root systems and vertical structure, or the influence of specific plant functional types). Nevertheless, given the current development of large-scale soil erosion modelling methods and available data (Orgiazzi and Panagos 2018), these were excluded from this analysis. In addition, we did not include land management practices directly in our model. This is mainly due to the focus of the paper on understanding the direct role of vegetation (which can account for land cover changes) on the supply of the soil protection service, and also on the absence of comparable land management practices data at the global scale [i.e., the same land-use type (e.g., agriculture) can have very different representations in Angola and in The Netherlands, thus including this factor would require fine scale data (Panagos et al. 2015b)]. Yet, even if estimated based on land cover information, this data would be introduced as a static variable in the model with little influence on the vegetation-driven patterns of soil protection. In general terms, the conceptual framework used here assumes that the ecosystem service supply is calculated from the difference between the potential erosion risk and the actual (or modeled) soil erosion using different components (Guerra et al. 2014, 2016a; Pinto-Correia et al. 2016):

(i) The potential soil erosion risk (*Y*);
(ii) Soil erosion protection, also referred as the capacity for ecosystem service supply (*e_s_*);
(iii) The protected soil, related to the ecosystem service supply (*Es*); and finally,
(iv) The soil erosion risk, also referred as remaining impact (*Be*).

The potential soil erosion risk is here defined as the total amount of soil erosion that would occur when, in a given place and time, vegetation is absent and therefore no ecosystem service is supplied by vegetation. Following the Universal Soil Loss Equation (USLE), this is a function of rainfall erosivity (i.e., the erosive potential of rainfall), soil erodibility (resulting from a combination of intrinsic soil properties) and local topography (Wischmeier and Smith 1978a, b; Yang et al. 2003; Ribeiro et al. 2004; Panagos et al. 2011) given by the equation:

(1) Y=R×LS×K,

where *Y* corresponds to the potential soil erosion risk, *R* to the rainfall erosivity, *LS* to the parameter related to the influence of local topography, and *K* to soil erodibility. A more extended description of the methods used to calculate each variable is given below.

Following the same conceptual framework, ecosystem service supply was calculated using the following equation:

(2) Es=Y−Be,

where *Es* corresponds to the protected soil, *Y* corresponds to the potential soil erosion risk, and *Be* to the modeled soil erosion risk, calculated based on:

(3) Be=Y×α,

where *Be* corresponds to modeled soil erosion, *Y* corresponds to the potential soil erosion risk, and α to a model parameter calculated as α = 1 − *e_s_* (where *e_s_* corresponds to the soil erosion protection parameter calculated here as the fraction of vegetation cover in a given pixel and moment in time; corresponding to the C-Factor in the USLE equation).

### Estimating the universal soil loss equation parameters

Vegetation cover (C-factor) was calculated as the fraction of green vegetation present in each pixel (Guerra et al. 2016a; Filipponi et al. 2018) (~ 1 km^2^ resolution in the equator using MODIS/Terra with monthly temporal resolution). The information for this factor has historically been derived from field experiments considering different aspects (Renard et al. 1997): (i) prior land use; (ii) soil cover by plant canopy; (iii) soil cover by crop residues; (iv) soil surface roughness; and (v) soil moisture. The evaluation of each aspect in a global framework is difficult because of the many possible combinations (Schönbrodt et al. 2010; Vanacker et al. 2014; Dutta 2016).

Here, we used the dataset provided by Filipponi et al. (2018) that calculated fractional green vegetation cover at the pixel level since land cover types (e.g., forest areas vs agricultural areas) were not differentiated in the calculation of vegetation dynamics. To generate the fractional vegetation cover layer (varying from 0 to 1), a dynamic masking procedure was created to remove pixels that had a low radiometric quality from each time-step (Small). For this step, the MODIS quality and pixel reliability flags were used to eliminate less reliable pixels (Hilker et al. 2015). Then, global spectral endmembers were selected from a representative temporal subset [using 2001 as a reference year (Zhang et al. 2015)] of global MODIS Terra acquisitions, transformed using principal component analysis to compress the radiometric information into fewer bands while keeping the pixel values’ variability (Valentini et al. 2015).

Finally, a linear spectral mixing analysis was applied for all time-steps, producing a monthly dataset of fractional green vegetation cover together with the estimation of the root-mean-square errors at the pixel level. To overcome the missing values generated by the dynamic masking, a “data interpolating empirical orthogonal function” (DINEOF) methodological approach was used to reconstruct missing data in the dataset’s multi-temporal series (Beckers et al. 2003). Missing pixels representing snow and ice cover, according to the MODIS quality flags, were not replaced by filtered values. The final product is obtained as a ratio of the pixel area from 0 to 1. The entire procedure regarding the calculation of the fraction of vegetation cover is further described in Filipponi et al. (2018). Notably, we did not make any correlation to any land-cover type since we calculated the fraction of vegetation cover directly from satellite imagery (in this case MODIS). This option was taken to avoid introducing another source of uncertainty and subjectivity in this global assessment (Van der Knijff et al. 2000; Grimm et al. 2002, 2003; Verheijen et al. 2009; Prasuhn et al. 2013).

Monthly surface precipitation was obtained from the CHELSA climate dataset (version 1.1) (Karger et al. 2016, 2017). CHELSA is a high resolution (see Table 1 for more details) climate dataset for the Earth land surface areas. It includes monthly and annual mean precipitation patterns from 1979 through 2013. It is based on a quasi-mechanistical statistical downscaling of the most recent global atmospheric reanalysis (ERA-interim) circulation model (Dee et al. 2011) with a GPCC [Global Precipitation Climatology Center, (Schneider et al. 2013)] and GHCN [Global Historical Climatology Network, (Peterson and Vose 1997; Lawrimore et al. 2011)] bias correction. CHELSA shows similar performance as high resolution satellite products such as Tropical Rainfall Measuring Mission (Goddard Space Flight Center Distributed Active Archive Center (GSFC DAAC) 2011) with the advantage of being globally available for a 35-year timeframe (Karger et al. 2017). It also includes topographic wind effects on precipitation and can distinguish between windward and leeward sites of an orographic barrier, as well as dry valleys (Karger et al. 2017).

Rainfall erosivity is calculated from rainfall amount and intensity (Wischmeier and Smith 1978a, b). In order to overcome the obstacle of estimating regional values without sufficient long-term records of rainfall intensity, past studies have tried to establish relationships between rainfall erosivity and available precipitation data, such as monthly and annual total precipitation (Renard and Freimund 1994; Renard et al. 1997). We used a maximization of the following two equations (Eqs. 4 and 5) to produce an erosivity map of each year (Yang et al. 2003):

(4) $$R=0.7397\times F^{1.847},$$

(5) $$R=95.77-6.081\times F+0.4770\times F^{2},$$

where *R* is the rainfall erosivity, and *F* corresponds to the Fournier index (Renard and Freimund 1994).

We assumed that this approach might introduce some errors for regions that have climate characteristics different from those of North America, but it offers a uniform temporally-explicit standard for evaluating rainfall erosivity across the globe. We used two equations simultaneously (with a maximization function) to overcome part of the issues raised by Naipal et al. (2015) regarding the over- and underestimation of erosivity given by the Renard and Freimund (1994) equation. To temporally disaggregate the rainfall erosivity values into a monthly distribution, we calculated the proportion of rainfall for each month of each year and then multiplied this proportion by the value of rainfall erosivity for each year. By doing this, we obtained a monthly disaggregation of the rainfall erosivity variable.

Soil erodibility represents the average long-term soil and soil-profile response to the erosive reaction to the processes of soil detachment and transport by raindrop impact and runoff (Yang et al. 2003). Consequently, this factor is best obtained from direct measurements on natural plots (Kinnell 2010), although this proves to be an unfeasible task on national or continental scales (Panagos et al. 2014). To overcome this issue, we used the soil properties that are most closely correlated with soil erodibility (i.e., soil texture, content of organic matter, soil structure and permeability) (Hengl et al. 2014; Batjes 2016), and calculated this variable based on the relationship proposed by several authors (Wischmeier and Smith 1978b; Renard et al. 1997; Panagos et al. 2014; Borrelli et al. 2017):

(6) $$K=\left[\frac{\left(2.1\times 10^{-4}\times M^{1.14}\times \left(12-a\right)+3.25\times \left(b-2\right)+2.5\times \left(c-3\right)\right)}{100}\right]\times 0.1317,$$

where *K* corresponds to soil erodibility expressed as Mg ha h MJ^−1^ ha^−1^ mm^−1^, *a* is the content of organic matter, *b* the soil structure parameter, *c* the profile permeability class (estimated based on both soil type and related properties), and *M* is the parameter related to soil texture. To allow for consistency across the globe, we used soil information regarding the top (0–30 cm) soil layer (corresponding to soil horizons A and E) available in the ISRIC World Inventory of Soil Emission Potentials (Hengl et al. 2014; Batjes 2016).

To account for the influence of topography and hydrology, a global scale ~ 0.25 km resolution elevation dataset (Table 1) was used the following equation (Moore and Burch 1986):

(7) $$LS={\left(\frac{a\times p}{22.13}\right)}^{0.4}\times {\left(\frac{sinsin\left(d\right)}{0.0896}\right)}^{1.3},$$

where *LS* represents the topographic factor (adimensional), *a* refers to a global scale flow accumulation model obtained from the elevation dataset, *p* to the pixel size, and *d* to the elevation slope in degrees.

A final spatial masking step was added to all inputs and outputs to exclude urban and water areas by identifying them using the dataset GlobeCover 2009 (see Table 1) and removing them from all layers using an overlay procedure. Due to their different spatial and temporal resolutions, the datasets were spatially harmonized. Although the output resolution is ~ 1 km (at the equator), all initial processing steps were implemented using a ~ 0.25 km spatial resolution matching the one of the topographic dataset (Table 1). This processing resolution implies that for all datasets with lower resolution, a disaggregation step had to be included in the processing chain. This step was implemented by disaggregating each 1 km pixel into a 4 × 4 grid of 0.25 km without altering the original values. The reverse procedure (from 0.25 to 1 km) followed a different approach as it was conducted at a later stage of the processing chain. For the aggregation of the values at 1 km^2^, we calculated the median value of all 0.25 km^2^ pixels contained in each 1 km^2^ pixel of the final output for each variable.

### Accounting for climate and vegetation effects

**Fig. 1:**
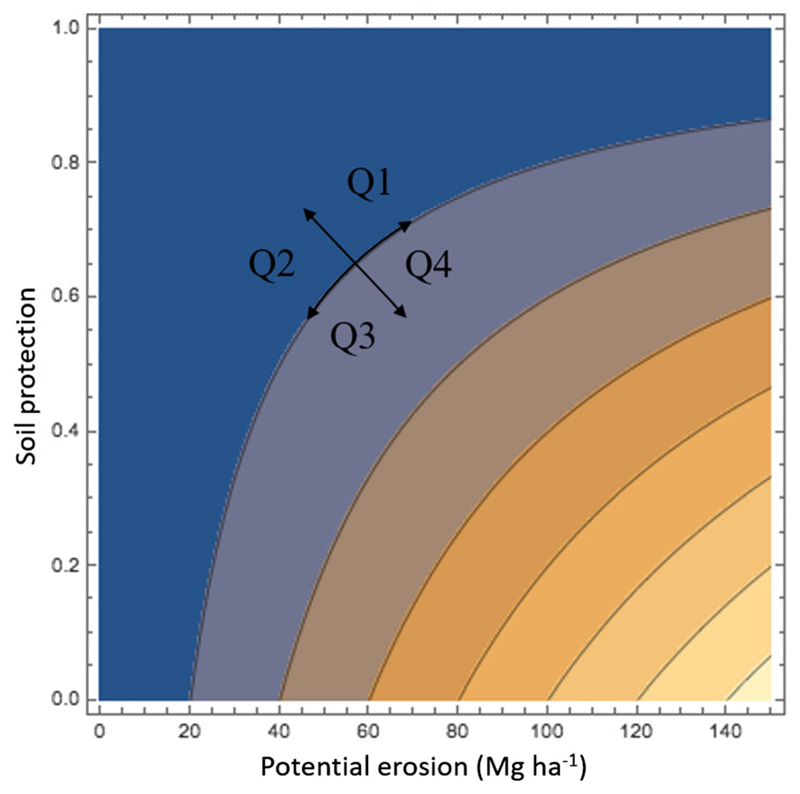
Contour plot of Eq. 3 comparing the effects of soil protection (vegetation driven, y-axis) and potential erosion (climate driven, x-axis) on soil erosion risk (colour pallet from blue [low erosion] to yellow [high erosion]). The two double arrow axes represent the potential changes in a point in space. Maximum erosion change is obtained by moving along the perpendicular axis to the level curve, no change in erosion is obtained by moving along the axis on the level curve. Q1, Q2, Q3, and Q4 represent the quadrants in Fig. 4a

To account for climate and vegetation effects, we made a comparison between the variation in soil protection and in soil erosion between 2001 and 2013. Since changes in soil erosion risk dynamically depend on changes in rainfall erosivity and on vegetation cover, an increase in soil erosion risk in an area with growing vegetation cover depicts an even higher increase of rainfall erosivity which the system could not cope with. Given that erosion risk is a function of soil protection and potential erosion (Eq. 3), we used a contour plot (Fig. 1) to analyse the effects of soil protection (vegetation driven) and potential erosion (climate driven) on erosion risk. Using the level curves depicted in Fig. 1, we are able to understand the direction of maximal change of erosion risk. This change occurs along the vector that is perpendicular to the level curve. Any variation along the axis on the level curve results in a zero change in soil erosion risk. This formulation allowed us to discriminate between positive (Q1 and Q2) and negative (Q3 and Q4) effects of soil protection (here represented by vegetation cover) and climate (here represented by rainfall erosivity) across the calculated domain.

### Vulnerability of soil communities to erosion

We selected two of the most comprehensive global soil biodiversity datasets, soil fungi (Tedersoo et al. 2014) to macrofauna (Mathieu and Lavelle 2016), in order to cover a wide range of soil biodiversity. For soil fungi, the dataset was generated based on the analysis of natural communities collected from 365 sites across the world using a uniform sampling protocol. Subsequently, these samples were interpolated using taxonomic richness of all fungi and an inverse distance weighted interpolation (IDW) algorithm that accounted for the relationship with mean annual precipitation (Tedersoo et al. 2014). The macrofauna dataset was developed in the context of the global soil biodiversity atlas (Orgiazzi et al. 2016) and represents the number of co-occurring soil macrofauna groups in five 25 × 25 cm^2^ × 30 cm deep samples, measured in each location at the same time, usually during the period with the peak of abundance. The macrofauna dataset includes 14 different groups (earthworms, ants, termites, spiders, millipedes, centipedes, isopods, fly larvae, cockroaches and mantids, moth and butterfly larvae, grasshoppers and crickets, gastropods, beetles, and other soil macrofauna) represented across 840 sites (corresponding to 2163 observations). This dataset was obtained using a species distribution model for each group in relation to a set of bioclimatic variables, land cover and altitude (Mathieu and Lavelle 2016).

Soil biodiversity data is often impaired by the lack of taxonomic and spatial representativeness that implies low resolution of the data and high uncertainty, particularly at the global scale (Cameron et al. 2018). Nevertheless, the datasets used describe a high taxonomic range, from fungi to macrofauna. Although a higher taxonomic depth as well as better spatial representation would improve both the conclusions derived from our results, these represent some of the best available information on global soil biodiversity (Cameron et al. 2019). Given these limitations, our approach only allows us to assess the potential vulnerability of soil biodiversity and not to go further and discriminate between taxonomic and functional groups. These two datasets cover a wide range of conditions from 0 to 2873 m in elevation (3 to 2259 in the case on macrofauna), from very low (0.12 [0.17 in the case of macrofauna]) to high (13.19 [6.58 in the case of fungi]) carbon content, to cold and dry annual conditions (− 0.2 °C; 246 mm [− 8 °C; 345 mm in the case of fungi]) to very warm and rainy conditions (29 °C; 4410 mm [29 °C; 3816 mm in the case of macrofauna]). With this range, most of the world conditions are covered by these datasets with the remaining gaps being located in desert/semi-arid zones and the northern Polar Regions.

Using these datasets, we calculated the differences in global soil erosion risk between 2001 and 2013 to identify cells that depict an increase in risk and aggregated the dataset to match the resolution of these first two datasets by using the median value of the aggregated cells. Finally, we did a pairwise comparison by overlaying both soil biodiversity datasets with the one for soil erosion increase. This comparison allowed us to illustrate the range of combinations between the increase in soil erosion risk and biodiversity and to further identify vulnerable areas, i.e., areas with high soil biodiversity that are potentially affected by areas with a higher increase in soil erosion risk.

## Results

### Global soil protection in space and time

**Fig. 2:**
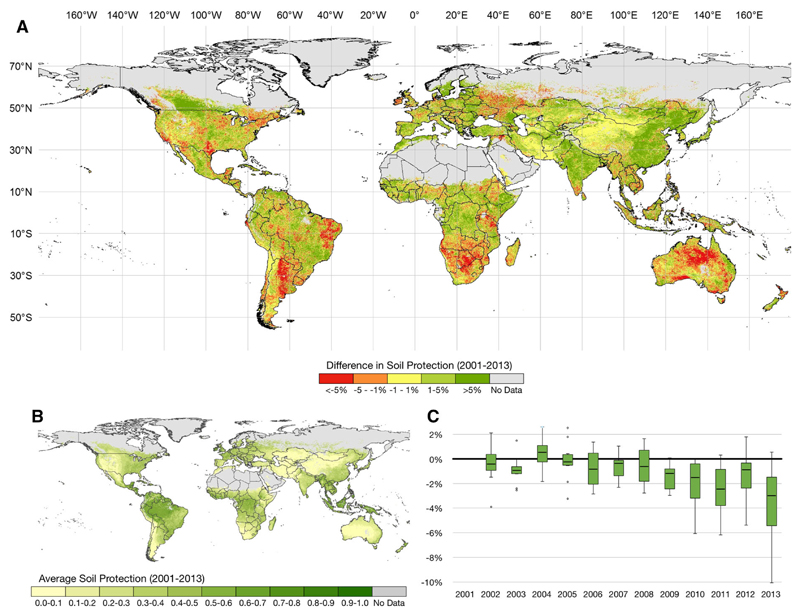
Soil erosion protection between 2001 and 2013: **a** relative differences between 2001 and 2013 (data available in https://figshare.com/s/d7918be095b8794f8eed); **b** spatial distribution of the average soil erosion protection for the period 2001–2013; c temporal distribution of the global ratio between the average soil erosion protection per terrestrial biome (Dinerstein et al. 2017) relative to 2001 [the values represent the relative differences of the ratio between the soil erosion and potential soil erosion (in %), and the within year distribution corresponds to the set of terrestrial biomes considered (see Fig. 5 for details)]

The patterns of soil erosion protection varied significantly across space and time, with extensive areas of the Southern hemisphere losing capacity to protect the soil over time (Fig. 2a). These losses are particularly evident in Argentina, Brazil, Australia, and in a number of South African countries like South Africa, Botswana or Namibia, coinciding with regions with relatively high rainfall erosivity (Panagos et al. 2017). At the same time, Ireland and the south of the United States of America are also particularly affected in their capacity to protect their soils. Overall, Central and Western Asia show the lowest values in soil erosion protection, followed by North Africa, Oceania, and North-East and South-East Asia. All these regions experienced an increase in soil erosion between 2001 and 2013 (see Online Appendix).

**Fig. 3:**
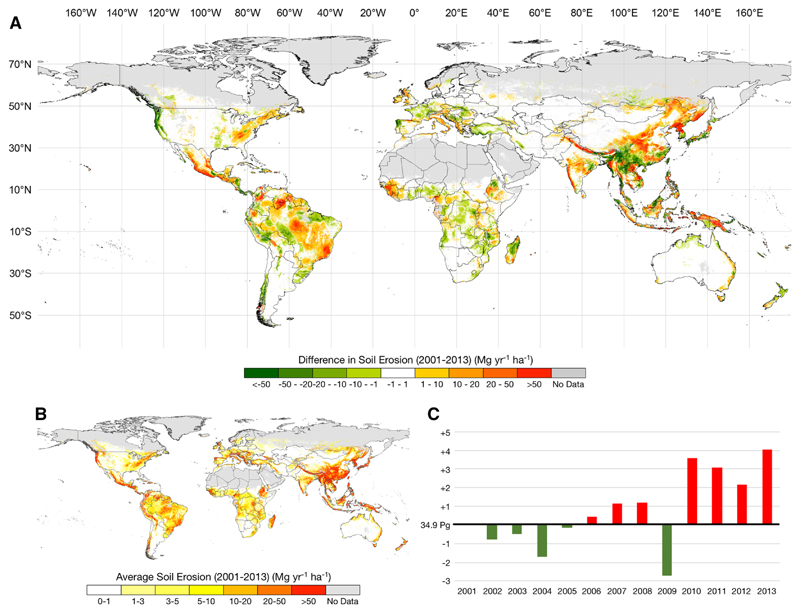
Soil erosion between 2001 and 2013: **a** spatial differences between 2001 and 2013; **b** spatial distribution of the average soil erosion between 2001 and 2013 (data available in https://figshare.com/s/db3d00d7c6bf657246c0); **c** temporal distribution of the global total soil erosion, values represent the difference between any given year and the total soil erosion of 2001 (34.9 Pg; green bars represent a decrease in soil erosion and red bars represent an increase in soil erosion)

Considering the temporal distribution of soil protection, between 2001 and 2013, there is a reduction of global soil protection by ~ 2.6%, although several regions in the globe have experienced opposite trends. This reduction is statistically significant for the years 2003 and after 2009 with an exception for 2012 (2-tailed t-test). This reduction in global soil protection appears to be a systematic negative trend across all terrestrial biomes considered in this study (Fig. 2c). This negative trend is mostly driven by changes in flooded grasslands and savannas (− 10.1%), temperate grasslands and savannas (− 6.6%), Mediterranean regions (flooded grasslands and savannas (− 5.5%) and in temperate broadleaf and mixed forests (− 5.3%). Globally, soil erosion rates are predominantly below 5 Mg year^−1^ ha^−1^ (66%) with increases in mountain areas and in areas with higher precipitation. Here we found a global overall increase of 11.7% in soil erosion rates between 2001 and 2013 (Fig. 3).

Overall, within the spatial scope of this paper, 55.4% of the globe registered an increase in soil erosion, with 11.2% of terrestrial surface above 1 Mg ha^−1^. In comparison with soil erosion protection, soil erosion risk systematically increased between 2006 and 2013 in relation to the baseline year (2001), with the exception of 2009 (Fig. 3c). This increase is particularly evident in temperate and tropical regions in South and Central America and in Asia, where in some cases the increase was higher than 50 Mg year^−1^ ha^−1^ (Fig. 3a). Although Asia remains one of the areas in the world with high soil erosion rates (Fig. 3b), our results show that while some areas have increased soil erosion rates, extensive areas in the south of China have significantly reduced their soil erosion risk. Other examples are found in South-East Asia (see Online Appendix). This reduction is driven by an increase in vegetation cover (Forzieri et al. 2017) (Fig. 2b) as well as a decrease in potential soil erosion risk driven by rainfall erosivity. Loss of vegetation cover also accounts for significant increases in soil erosion risk in South America, particularly in areas where deforestation is a main driver.

### The vulnerability of soils and soil biodiversity

**Fig. 4:**
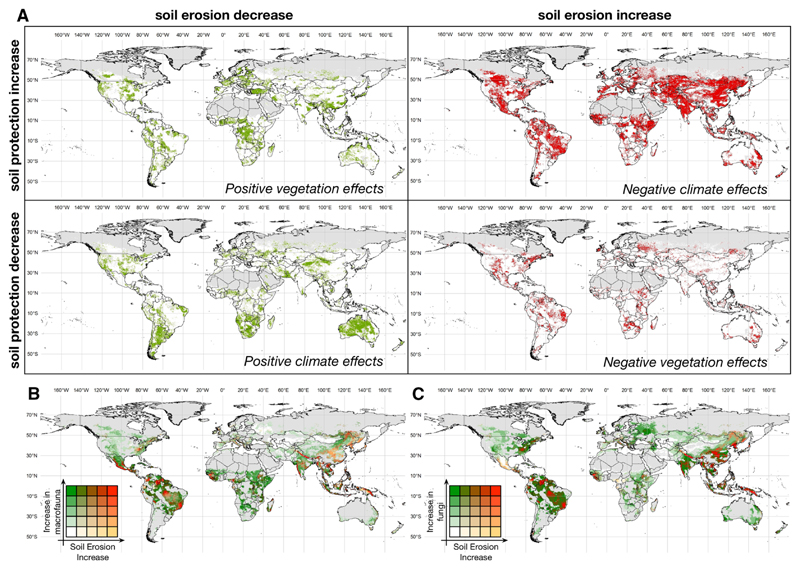
Spatial segmentation of the world according to the variation in soil protection and in soil erosion (between 2001 and 2013) (**a**), and the relation between the variation of soil erosion (between 2001 and 2013) and soil macrofauna (**b**) and fungi (**c**). In **a**, the quadrants represent the areas classified from positive to negative vegetation and climate effects. This classification was done by assessing each pixel as having increased or decreased in the period between 2001 and 2013 and classifying them accordingly (e.g., Q1 corresponds to areas that show and increase in soil protection [driven by vegetation] and a decrease in soil erosion). Q1 and Q3 represent predominant effects of vegetation cover, i.e., where vegetation cover has a stronger effect irrespectively of climate dynamics. The quadrants Q2 and Q4 represent predominant effects of climate, i.e., where climate (here reflecting rainfall erosivity) has a stronger effect irrespective of the vegetation cover dynamics. For **b** and **c**, only areas with increases in soil erosion were used (according to Fig. 3a); and for soil biodiversity (macrofauna and fungi), the distribution data was classified into five classes according to a quantile distribution. Red areas depict pixels with high soil biodiversity and high soil erosion change, while white areas depict pixels with low soil biodiversity and low soil erosion change

By comparing the spatial distribution of the temporal difference between 2001 and 2013 of both soil erosion protection and soil erosion risk (Fig. 4a), we were able to discriminate between relevant climate and vegetation effects across the globe. Although indirect effects of climate on soil erosion risk (i.e., through effects on vegetation dynamics) may have a role in this distinction, the separation method used only accounts for direct climate or vegetation effects. In this context, it is important to note that the global effects on soil erosion risk are predominantly climatic (66.1% of the land surface assessed). Vegetation cover effects only account for 33.9% of the land surface assessed, with 63.6% of these effects being positive effects, i.e., reflecting a reduction of local soil erosion risk related to an increase in vegetation cover independently of climate dynamics (Forzieri et al. 2017).

Climate accounted for 51.6% of the area of risk reduction (i.e., reflecting the reduction of soil erosion caused primarily by a decrease in rainfall erosivity independently of vegetation dynamics) and for 77.7% of the area where soil erosion risk has increased. These results illustrate the vulnerability of soil resources to significant changes in climate. Although land cover and land use change represent an important global change driver, our results show that accounting for climate change effects is crucial not only to better understand the mechanistic processes behind global soil erosion risk and protection, but also to design adequate regional- and national-level policy solutions for soil protection.

By examining the overlap the spatial patterns of soil erosion risk with those for soil macrofauna, we identified that Central and South America (particularly Brazil, Venezuela, and Mexico), West Africa (particularly Guinea, Sierra Leone, and Liberia), and Asia and the Pacific (particularly India, China, Nepal, North Korea, Indonesia, and Papua New Guinea; Fig. 4b) are areas where soil macrofauna was potentially more affected by soil erosion. These vulnerable areas are also consistent with the ones for soil fungi (Fig. 4c).

Globally, the most vulnerable areas, i.e., areas with high soil biodiversity and increased risk, correspond to 6.4% and 7.6% for macrofauna and fungi, respectively. These areas of higher predicted vulnerability mostly coincide with areas of negative climate impacts (Fig. 4), which underlines the need for specific climate mitigation measures that allow the effects of increase rainfall erosivity to be overcome.

## Discussion

Our results show a high spatial and temporal variability in soil erosion protection due to climate dynamics and changes in vegetation cover, with expected higher values for the Tropical, Mesoamerican and East European regions (Fig. 2a; Online Appendix). Globally, and following the patterns of precipitation (see Online Appendix), lower average values of soil erosion protection are found in the Southwest and Northwest of the American continent, in South Africa, Central Australia and in Central Asia. Although higher values of vegetation cover are related to a higher soil protection capacity, it is important to note that these do not reflect entirely the dynamics of soil erosion risk since the relation between erosion and vegetation cover is not linear (Guerra et al. 2014). Because vegetation, particularly natural vegetation, is in many systems dependent on the availability of water, fluctuations according to precipitation patterns are expected. Other studies also show that land management (Guerra and Pinto-Correia 2016; Borrelli et al. 2017; Steinho and Burkhard 2018) or extreme events (Hosseini et al. 2016) are critical to the dynamics of soil erosion protection, but this was not directly assessed within this study. While not addressing these aspects directly we acknowledge that we may be underestimating their effects on soil erosion rates, particularly in regions that have experienced several of these events (e.g., SW of North America).

In the case of soil erosion protection, we show a decrease in soil protection between 2001 and 2013 (Fig. 2a, c) that, when focussing on the distribution within the terrestrial biomes, is not correlated with the changes in soil erosion rates. This mismatch is mostly related to climate, particularly the reduction in precipitation that, in turn, reduced the relative amount of soil erosion in a given place and time. Although potentially positive for soil conservation, a reduction in precipitation can have negative implications for (i) soil biodiversity, by reducing the soil water content and thus affecting the dynamics, biomass, and diversity of soil organisms (Coleman et al. 2004), and (ii) other soil processes, by changing humidity and potentially changing the rates of soil decomposition (Djukic et al. 2018). These phenomena can lead to a large-scale degradation of the landscape and both local (Van Oost et al. 2000; Harmon and Doe III 2001) and regional levels (Guerra et al. 2016a, b).

On the other hand, soil erosion increased in the same period by 11.7% (Fig. 3c). This increase affected mostly South and Central America and Asia, i.e., regions which were already affected by high rates of soil erosion (Fig. 3b). These results are supported by several regional studies. Some of these studies report no change, or decrease in erosivity in NW Mediterranean areas (Angulo-Martínez and Beguería 2012; Beguería et al. 2018; Serrano-Notivoli et al. 2018), but also the opposite trend for Chinese landscapes (Xin et al. 2011). These trends identified by regional studies are in line with the ones found by our study. These landscape-level changes can lead to the loss of arable soils (e.g., leading to potential conflicts as seen recently: Parolari et al. 2016) and an increase of environmental conflicts (Diehl 2018).

Supported by recent studies that follow a similar modelling approach (Borrelli et al. 2017), the erosion rates reported here are at least two times lower than other values previously reported in the literature. This difference is in part explained by the unprecedented way in which Borrelli et al. (2017) and the present study, which further integrated global dynamics of rainfall and vegetation cover, were able to map and study this phenomenon. Nevertheless, further validation of absolute erosion values by including soil erosion rates systematically collected across the globe in a coherent modelling framework is needed. These findings are in line with a recent meta-analysis of globally distributed soil erosion rates (García-Ruiz et al. 2015), which reported a systematic over-estimation of previous model-based approaches in comparison to measured soil erosion rates. Increasing the systematization and availability of experimental and local data on soil erosion together with meta-information on the conditions leading to the event (e.g., climatic, management, cover) is critical to extend this assessment further (García-Ruiz et al. 2015).

Nevertheless, such temporally explicit models of soil erosion as well as soil protection are key to understand global cycles (Chappell et al. 2015), but also to support current environmental and land degradation target in the scope of the United Nations Land Degradation neutrality targets and the Convention to Combat Desertification (IPBES 2018). Regarding the later, by showing the global patterns of vulnerability of soil communities to soil erosion, we provide context for the identification of target areas where soil biodiversity conservation is needed the most. As a result, regions like Southeast Asia and the South American tropics should be prioritized in this effort, particularly in the context of the current push for afforestation (Crowther et al. 2015, 2018, 2019). Nevertheless, when designing landscape policies, decision-makers should consider the implications of such land-change dynamics not only as a direct effect on soil erosion, but also on biodiversity and on the character of the landscape itself (Westoby 1987; Frank et al. 2014; Blaikie 2016).

### Cross biome comparison

**Fig. 5:**
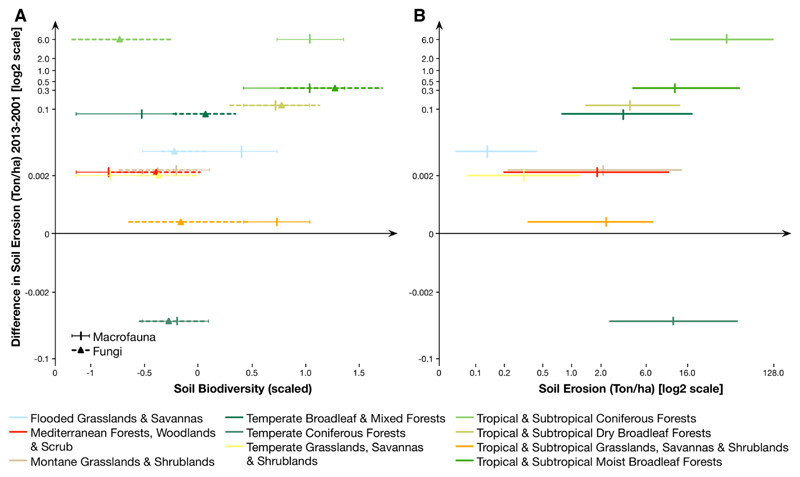
Pairwise relation between the difference in soil erosion from 2001 and 2013 (y axis) and **a** soil biodiversity (the values on the x-axis represent scaled values of the biodiversity layers used in Fig. 4) and **b** average soil erosion between 2001 and 2013. The central point represents the median value for both axes and the horizontal bars represent the 1st and 3rd quantiles, respectively for each biome represented

When compared across biomes, the match between the changes in soil erosion rates and soil biodiversity becomes more apparent (Fig. 5b). Here, we compared the expected changes in soil erosion (2001–2013) with the scaled values of soil biodiversity (Fig. 5a) and the average soil erosion for the period (Fig. 5b). This comparison allowed us to explore the potential vulnerability of the soil biodiversity of specific biomes (i.e., bigger increases in soil erosion in biomes with higher diversity) but also identify which biomes (with comparable erosion rates) have experienced stronger changes (e.g., Mediterranean forests and Tropical and subtropical grasslands show similar erosion rates but quite different increases in soil erosion). With some exceptions (e.g., temperate coniferous forests), the temporal increase in soil erosion is more pronounced in biomes with higher soil erosion rates, with potential causal effects on the reduction in soil fertility and in soil aggregate stability that will, in turn, increase farming inputs and reduce the benefits that people obtain from soils and soil communities (Wall and Six 2015).

Overall, soil erosion prevention represents an important factor for soil conservation as it affects fertility rates and reduces the capacity of soils to sustain above- and below-ground biodiversity (Orgiazzi and Panagos 2018). Across the globe, our study also shows that the soil communities affected can be quite different in biodiversity. While in some cases (e.g., temperate grasslands, savannas and shrublands), both macrofauna and fungi present similar relative biodiversity; for other biomes (e.g., tropical and subtropical coniferous forests), there are significant differences between the two groups of soil organisms (Fig. 5a). Given that these communities simultaneously are potentially affected by soil erosion and can provide more sustainable conditions and support the reduction of soil erosion rates (Wall et al. 2012), further study is needed to understand their compositional structure and to effectively identify their vulnerability to this process. The present results suggest that soil erosion may represent a major threat to a significant portion of terrestrial biodiversity (Cameron et al. 2018) at various locations of the globe. Simultaneously, this change in soil biodiversity is likely to also have strong feedback effects on many critical ecosystem processes (Jing et al. 2015; Delgado-Baquerizo et al. 2016; Soliveres et al. 2016; Trogisch et al. 2017).

In parallel, sediment removal and transport can have large and lasting offsite-effects in rivers and channels in the affected regions by reducing their navigation potential, impacting fish communities and stocks, and by reducing water quality (Kondolf et al. 2014; Rickson 2014; Kjelland et al. 2015). Although not considered at the global scale, these mechanisms are critical at both regional and local scales. In this respect, it is important to note that most of the areas that showed an increase in soil erosion are also areas with high freshwater diversity (Tedesco et al. 2017). Therefore, conserving soil and reducing soil erosion has to go beyond farming and crop production and extend to the realm of nature conservation. Reducing soil erosion not only has a local positive effect on soil biodiversity and soil ecosystem processes, but also has the potential to have important cascade effects on other terrestrial and aquatic ecosystems (Pimentel and Kounang 1998; Powlson et al. 2011).

### Model caveats

In most cases, to obtain estimates of soil erosion prevention and soil erosion, it is necessary to use models that support the prediction of areas where surveys are not available or are not possible. Although these models inform our understanding of the spatial and temporal patterns of soil erosion and protection, they often lack of proper validation datasets for large-scale assessments. Another source of uncertainty is the lack of local parameterization of empirical models. For instance, the relationships between different parameters influencing soil erosion may vary across regions or land cover types and these differences are often not considered when using empirical models (e.g., USLE).

The USLE is a purely deterministic model, in which the product of different variables is used to derive the amount of soil loss. In this case, a rigorous assessment of uncertainties is not feasible, nor would it be meaningful, unless the uncertainties of the input layers and their propagation are quantified (Borrelli et al. 2017). Furthermore, most of the variables used as input lack proper uncertainty assessments, which in turn limits the capacity to assess and propagate errors across the modelling framework. Here, our main focus was to explore trends and temporally explicit relative differences without focussing on absolute values, which models often overestimate (García-Ruiz et al. 2015). At the same time, many areas across the globe lack available data on erosion rates and sediment loads, adding to the uncertainty related to the implementation of soil erosion models.

Additionally, when comparing the changes in soil protection to the changes in soil erosion, we found a general spatial mismatch (Fig. 4a) i.e., decreases in soil erosion do not always match increases in soil protection but rather changes in climatic patterns. The direction and intensity of these climatic changes may also have indirect effects on soil biodiversity through changes in vegetation (Sylvain and Wall 2011). For example, the ecotone between taiga and tundra is moving northward due to global warming (Skre et al. 2002). This change will affect soil erosion as vegetation is changing here due to climate change, which will then affect soil biodiversity. This interaction makes the further understanding of the influence of climate and vegetation cover on the global patterns of soil erosion protection critical for policy formulation.

## Conclusions

These results illustrate the importance of climate mitigation measures for soil conservation. Irrespective of the importance of land cover change for overall global change, soil conservation policy should focus on the current and potential effects of climate on soil erosion as our results show that in many places of the world this is the main controlling factor of soil erosion. Given the difficulty of implementing large-scale effective soil conservation measures that mitigate the ever-growing effects of climate, it is important to promote integrated approaches that incorporate both the economic and conservation risks associated with the loss of soil. Globally, soil erosion is expected not only to have an impact on soil conditions but could also threaten soil biodiversity, with 6.4% (for soil macrofauna) and 7.6% (for soil fungi) of increased soil erosion risk areas also impacting regions with high soil biodiversity. These results indicate not only that an increasing proportion of soils are degraded globally, but also that many degraded regions coincide with high levels of soil biodiversity.

Although some attempts to merge soil biodiversity and erosion modelling have recently been made (e.g., Orgiazzi and Panagos 2018), these are mostly expert based parameter estimations rather than actual analyses of the reciprocal effects between diversity and erosion. Given the current limitations of soil erosion modelling, including these feedback effects has the potential to improve model estimates; allow for a better assessment of ecosystem service supply, including the estimating the role of soil biodiversity on these estimations; and further differentiate the vulnerability of soils to erosion. Going beyond expert knowledge approaches, our study calls for demonstrable erosion modelling ~ soil biodiversity integration either through experimental work or through the development of causal effects models that can account for these interactions.

In parallel, in a meeting of the Intergovernmental Technical Panel on Soils held in June 2018, member states defined understanding global soil erosion and soil biodiversity dynamics to support effective action as a critical priority for the coming two years. The relation shown here between soil biodiversity and the increase in soil erosion gives an initial view on the potential interactions of these two variables and on the potential vulnerability of soil biodiversity to soil erosion. Regarding the latter, our results stress the need for more global mechanistic approaches that combine soil biodiversity and soil processes in order to better understand soil dynamics in the face of global change drivers. Furthermore, in the context of climate change and the projected alterations in rainfall patterns worldwide, these mechanistic models and global assessments could play a vital role in identifying and anticipating future vulnerable areas. Finally, these global analyses can support the design of experimental settings under climate change, thus improving coordination among nations for the development of better global mitigation and/or adaptation solutions. Our results support these efforts by providing a standard and integrated assessment of soil erosion risk and ecosystem service supply.

## Supplementary Material

The online version of this article (https://doi.org/10.1007/s10980-020-00984-z) contains supplementary material, which is available to authorized users.

Supplementary file 3

Supplementary file 1

Supplementary file 2

## Figures and Tables

**Table 1:** Input reference datasets included in the process-based modeling

Dataset	Resolution at the equator	Temporal resolution	References
Global multi-resolution terrain elevation data 2010 (GMTED2010)	7.5 arc-sec (~ 0.25 km)	Static	Danielson and Gesch (2011)
Climatologies at high resolution for the earth’s land surface areas (CHELSA) v1.1	30 arc-sec (~ 1 km)	Monthly	Karger et al. (2017)
FCover—fraction of green vegetation cover	30 arc-sec (~ 1 km)	Monthly	Filipponi et al. (2018)
Soil grids	30 arc-sec (~ 1 km)	Static	Hengl et al. (2014) and Batjes (2016)
GlobCover 2009	9 arc-sec (~ 0.3 km)	Static	Bontemps et al. (2011)
