# Supplementary material for: Global vulnerability of soil ecosystems to erosion
Source: Landsc Ecol. Author manuscript; Available in PMC 2021 Apr 1. (PMC7316572; doi:10.1007/s10980-020-00984-z)
Supplement: Supplementary file 3 [file EMS86636-supplement-Supplementary_file_3.docx]

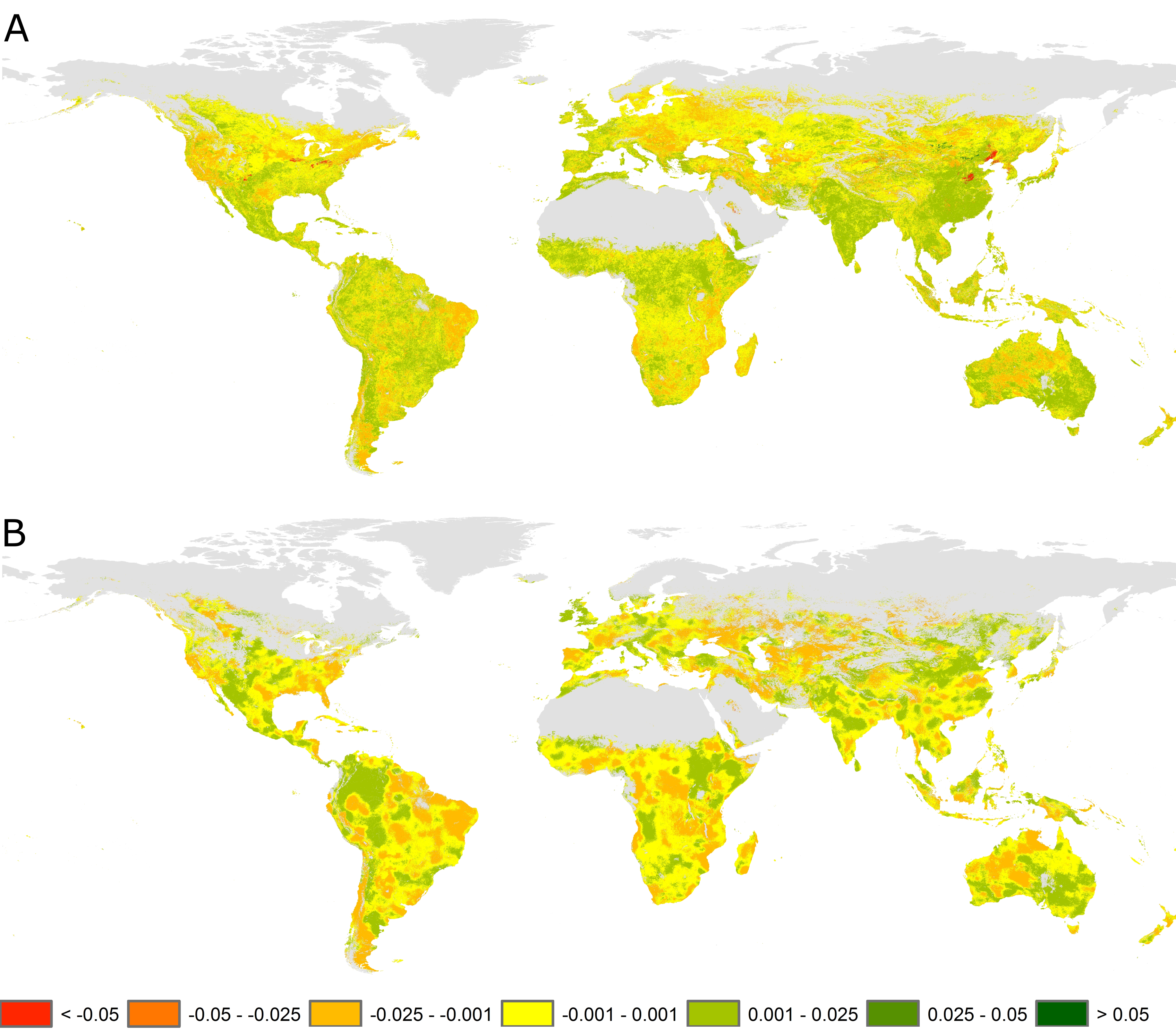


**Figure S1:** Linear regression (generalized least squares) of the temporal trend of the capacity for soil protection (A) and of soil protection (B). The linear regression was calculated at pixel level considering the entire temporal scope (2001-2013). Values represent the slope argument of the linear regression. Negative values indicate loss and positive values indicate gain.


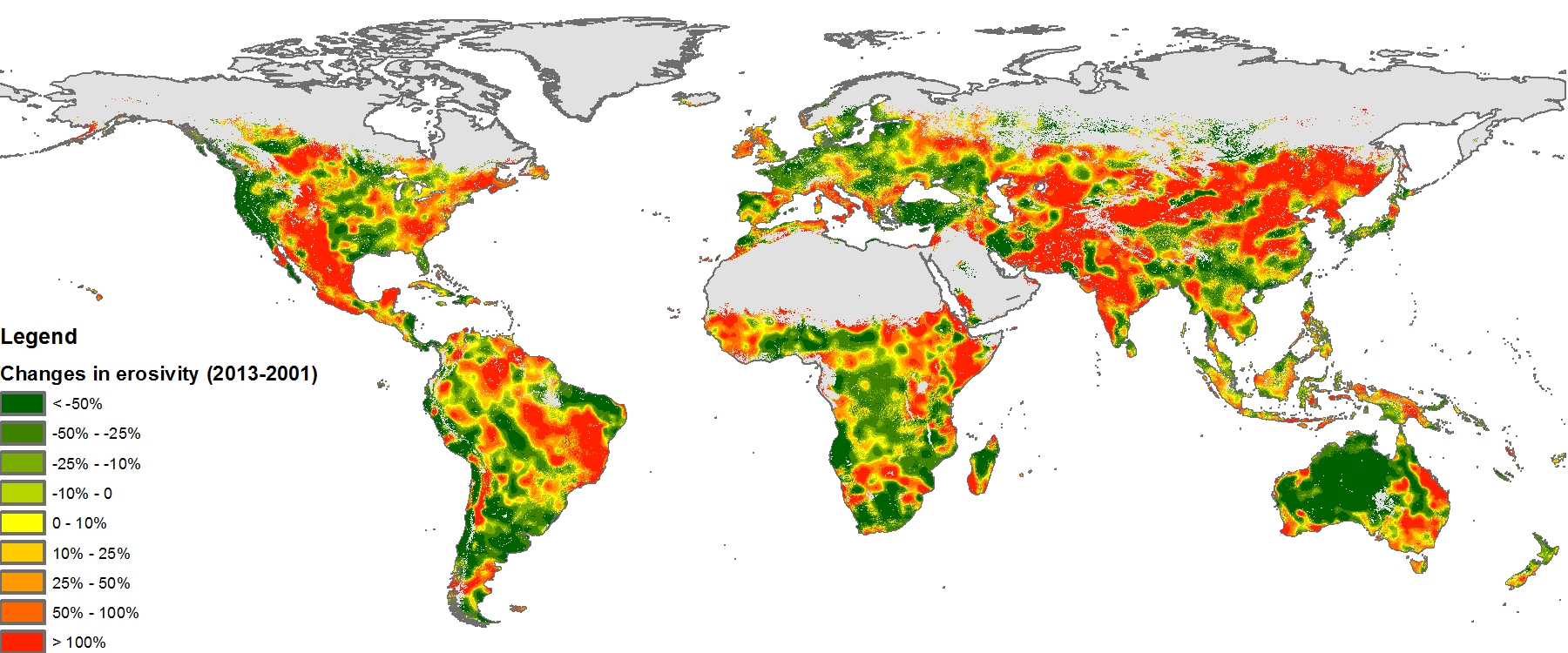


**Figure S2:** Changes in the erosivity patterns between 2001 and 2013.
